# Supplementary material for: Pyruvate Dehydrogenase Kinase 1 inhibition mediated oxidative phosphorylation enhancement in cartilage promotes osteoarthritis progression
Source: BMC Musculoskelet Disord. 2023 Jul 20;24:597. doi: 10.1186/s12891-023-06585-6 (PMC10357736; doi:10.1186/s12891-023-06585-6)
Supplement: Supplementary file 1 — Additional file 1. [file 12891_2023_6585_MOESM1_ESM.zip › KEGG.Gsea.1653622432464/KEGG_HISTIDINE_METABOLISM.html]

Details for gene set KEGG\_HISTIDINE\_METABOLISM[GSEA]

|  || Dataset | 55457\_collapsed\_to\_symbols.cls.cls #OA\_versus\_normal.cls.cls #OA\_versus\_normal\_repos |
| Phenotype | cls.cls#OA\_versus\_normal\_repos |
| Upregulated in class | OA |
| GeneSet | KEGG\_HISTIDINE\_METABOLISM |
| Enrichment Score (ES) | 0.6186523 |
| Normalized Enrichment Score (NES) | 2.1057427 |
| Nominal p-value | 0.0 |
| FDR q-value | 0.0016862552 |
| FWER p-Value | 0.006 |
Table: GSEA Results Summary

  

Fig 1: Enrichment plot: KEGG\_HISTIDINE\_METABOLISM      
 Profile of the Running ES Score & Positions of GeneSet Members on the Rank Ordered List

  

| SYMBOL | TITLE | RANK IN GENE LIST | RANK METRIC SCORE | RUNNING ES | CORE ENRICHMENT || 1 | BUD23 | BUD23 rRNA methyltransferase and ribosome maturation factor [Source:HGNC Symbol;Acc:HGNC:16405] | 210 | 0.813 | 0.0993 | Yes |
| 2 | HNMT | histamine N-methyltransferase [Source:HGNC Symbol;Acc:HGNC:5028] | 700 | 0.594 | 0.1458 | Yes |
| 3 | LCMT1 | leucine carboxyl methyltransferase 1 [Source:HGNC Symbol;Acc:HGNC:17557] | 735 | 0.585 | 0.2263 | Yes |
| 4 | HEMK1 | HemK methyltransferase family member 1 [Source:HGNC Symbol;Acc:HGNC:24923] | 742 | 0.583 | 0.3087 | Yes |
| 5 | LCMT2 | leucine carboxyl methyltransferase 2 [Source:HGNC Symbol;Acc:HGNC:17558] | 946 | 0.529 | 0.3682 | Yes |
| 6 | ALDH2 | aldehyde dehydrogenase 2 family member [Source:HGNC Symbol;Acc:HGNC:404] | 1003 | 0.517 | 0.4373 | Yes |
| 7 | ALDH3A2 | aldehyde dehydrogenase 3 family member A2 [Source:HGNC Symbol;Acc:HGNC:403] | 1263 | 0.465 | 0.4833 | Yes |
| 8 | TRMT11 | tRNA methyltransferase 11 homolog [Source:HGNC Symbol;Acc:HGNC:21080] | 1885 | 0.374 | 0.4883 | Yes |
| 9 | AOC1 | amine oxidase copper containing 1 [Source:HGNC Symbol;Acc:HGNC:80] | 1992 | 0.362 | 0.5315 | Yes |
| 10 | ALDH7A1 | aldehyde dehydrogenase 7 family member A1 [Source:HGNC Symbol;Acc:HGNC:877] | 2158 | 0.343 | 0.5675 | Yes |
| 11 | MAOB | monoamine oxidase B [Source:HGNC Symbol;Acc:HGNC:6834] | 2192 | 0.339 | 0.6132 | Yes |
| 12 | ALDH9A1 | aldehyde dehydrogenase 9 family member A1 [Source:HGNC Symbol;Acc:HGNC:412] | 2795 | 0.270 | 0.6048 | Yes |
| 13 | FTCD | formimidoyltransferase cyclodeaminase [Source:HGNC Symbol;Acc:HGNC:3974] | 3064 | 0.244 | 0.6187 | Yes |
| 14 | ASPA | aspartoacylase [Source:HGNC Symbol;Acc:HGNC:756] | 3610 | 0.192 | 0.6037 | No |
| 15 | ALDH3B2 | aldehyde dehydrogenase 3 family member B2 [Source:HGNC Symbol;Acc:HGNC:411] | 5617 | 0.022 | 0.4511 | No |
| 16 | ALDH1A3 | aldehyde dehydrogenase 1 family member A3 [Source:HGNC Symbol;Acc:HGNC:409] | 5831 | 0.007 | 0.4355 | No |
| 17 | ALDH3B1 | aldehyde dehydrogenase 3 family member B1 [Source:HGNC Symbol;Acc:HGNC:410] | 5862 | 0.004 | 0.4338 | No |
| 18 | ALDH3A1 | aldehyde dehydrogenase 3 family member A1 [Source:HGNC Symbol;Acc:HGNC:405] | 6413 | -0.036 | 0.3962 | No |
| 19 | METTL2B | "methyltransferase 2B, methylcytidine [Source:HGNC Symbol;Acc:HGNC:18272]" | 6522 | -0.044 | 0.3941 | No |
| 20 | ALDH1B1 | aldehyde dehydrogenase 1 family member B1 [Source:HGNC Symbol;Acc:HGNC:407] | 6551 | -0.046 | 0.3984 | No |
| 21 | HDC | histidine decarboxylase [Source:HGNC Symbol;Acc:HGNC:4855] | 6599 | -0.050 | 0.4018 | No |
| 22 | MAOA | monoamine oxidase A [Source:HGNC Symbol;Acc:HGNC:6833] | 8271 | -0.177 | 0.2971 | No |
| 23 | DDC | dopa decarboxylase [Source:HGNC Symbol;Acc:HGNC:2719] | 8605 | -0.202 | 0.2999 | No |
| 24 | HAL | histidine ammonia-lyase [Source:HGNC Symbol;Acc:HGNC:4806] | 9053 | -0.236 | 0.2987 | No |
Table: GSEA details [plain text format]

  

Fig 2: KEGG\_HISTIDINE\_METABOLISM      
 Blue-Pink O' Gram in the Space of the Analyzed GeneSet

  

Fig 3: KEGG\_HISTIDINE\_METABOLISM: Random ES distribution      
 Gene set null distribution of ES for **KEGG\_HISTIDINE\_METABOLISM**
